# The Dysregulated Galectin Network Activates NF-κB to Induce Disease Markers and Matrix Degeneration in 3D Pellet Cultures of Osteoarthritic Chondrocytes

**DOI:** 10.1007/s00223-020-00774-4

**Published:** 2020-11-13

**Authors:** K. M. Pichler, D. Weinmann, S. Schmidt, B. Kubista, R. Lass, L. Martelanz, J. Alphonsus, R. Windhager, H. -J. Gabius, S. Toegel

**Affiliations:** 1 Department of Orthopedics and Trauma Surgery, Karl Chiari Lab for Orthopaedic Biology, Medical University of Vienna, Waehringer Guertel 18-20, 1090 Vienna, Austria; 2 Institute of Physiological Chemistry, Faculty of Veterinary Medicine, Ludwig‐Maximilians University Munich, Munich, Germany; 3 Department of Orthopedics and Trauma Surgery, Division of Orthopedics, Medical University of Vienna, Vienna, Austria; 4 Ludwig Boltzmann Institute for Arthritis and Rehabilitation, Vienna, Austria

**Keywords:** Chondrocytes, Galectin, Lectin, NF-κB, Osteoarthritis, 3D cell culture

## Abstract

This work aimed to study the dysregulated network of galectins in OA chondrocyte pellets, and to assess whether their recently discovered activity as molecular switches of functional biomarkers results in degradation of extracellular matrix in vitro. Scaffold-free 3D pellet cultures were established of human OA chondrocytes. Expression and secretion of galectin(Gal)-1, -3, and -8 were monitored relative to 2D cultures or clinical tissue sections by RT-qPCR, immunohistochemistry and ELISAs. Exposure of 2D and 3D cultures to an in vivo-like galectin mixture (Gal-1 and Gal-8: 5 µg/ml, Gal-3: 1 µg/ml) was followed by the assessment of pellet size, immunohistochemical matrix staining, and/or quantification of MMP-1, -3, and -13. Application of inhibitors of NF-κB activation probed into the potential of intervening with galectin-induced matrix degradation. Galectin profiling revealed maintained dysregulation of Gal-1, -3, and -8 in pellet cultures, resembling the OA situation in situ. The presence of the galectin mixture promoted marked reduction of pellet size and loss of collagen type II-rich extracellular matrix, accompanied by the upregulation of MMP-1, -3, and -13. Inhibition of p65-phosphorylation by caffeic acid phenethyl ester effectively alleviated the detrimental effects of galectins, resulting in downregulated MMP secretion, reduced matrix breakdown and augmented pellet size. This study suggests that the dysregulated galectin network in OA cartilage leads to extracellular matrix breakdown, and provides encouraging evidence of the feasible inhibition of galectin-triggered activities. OA chondrocyte pellets have the potential to serve as in vitro disease model for further studies on galectins in OA onset and progression.

**Electronic supplementary material:**

The online version of this article (10.1007/s00223-020-00774-4) contains supplementary material, which is available to authorized users.

## Introduction

Osteoarthritis (OA) poses an enormous burden on the individual patient that adds up to a profound socioeconomic impact on health care sectors worldwide [1]. This justifies vigorous efforts to systematically study the molecular basis of disease onset and progression with the aim to develop innovative therapeutic strategies that interfere with the complex OA pathogenesis [2, 3], currently culminating in multi-omics approaches to identify relevant molecular signatures [4]. Obviously, the strategic combination of work on clinical material with tailored in vitro models would be helpful to generate meaningful pathobiological results and improve translatability of new knowledge into clinical practice [5]. From a practical perspective, two-dimensional (2D) cell cultures have been considered as the starting point on the way to mimic an in situ-like microenvironment in vitro*.* The step from 2D to three-dimensional (3D) systems, however, is the logical continuation to narrow the gap from in vitro to in vivo conditions [6, 7]. Since the status of 3D culture for joint degeneration has recently been assessed to be “still in infancy” [7], intensive research is urgently required to foster its maturation.

Following this reasoning, the applicability of scaffold-free 3D pellet cultures of OA chondrocytes needs to be rigorously put to the test. When starting such cultures with mesenchymal stem cells, lubricin-expressing chondrocytes were found to be generated [8], and postnatal chondroprogenitors were shown to produce zonally organized hyaline cartilage in pellet culture [9]. When using chondrocytes from cartilage regions that were unaffected from OA, distinct aspects of the mRNA expression profile associated with the chondrogenic potential and the hypertrophic phenotype revealed differences between cells in 2D and 3D cultures [10]. The hereby proven feasibility to grow pellets from mature chondrocytes prompted us to do so with cells from clinical OA specimens. Such 3D cultures could well be an appropriate model to answer the open question on whether the expression of OA biomarkers—that is typically studied in 2D cultures with cells isolated from clinical specimens—is of functional significance in the context of OA cartilage degeneration. Considering OA cartilage in vivo as standard, the comparative profiling of histological appearance and biomarker presence in pellets will yield decisive information on the status of pellets as OA model.

We here focus on a class of recently discovered potent enhancers of pro-degradative/inflammatory effectors, i.e., ga(lactose-binding)lectins (galectins). On a fundamental level, the members of the galectin family are emerging as versatile missing link between glycan-encoded signals presented by cellular glycoconjugates and manifold processes in cellular homeostasis and in diseases [11]. A context-specific multifunctionality is characteristic for galectins that share the β-sandwich fold and a sequence signature for specific ligand contact [12–14]. Initial studies in animal cartilage, mouse models, and human OA cartilage with galectins-1 and -3 (Gal-1 and -3) revealed evidence for their presence in cartilage and for their potential to affect pig chondrocyte differentiation and catabolic processes as well as for their association with chondrocyte survival and disease manifestation ([15, 16]; for recent review, please see [17]). Systematic mapping of galectin presence in vitro in human OA chondrocytes and in sections of OA lesions traced a significant dysregulation that was found to correlate with the histological degree of cartilage degeneration in the cases of Gal-1, -3, and -8 [18]. By subsequently studying each of these galectins in functional assays in vitro*,* we discovered (i) the glycan-inhibitable induction of an NF-κB-dependent increase of functional disease markers and (ii) first cues for a teamworking among these lectins [19–21].

The aim of this study was to evaluate the applicability of OA chondrocyte pellets as a functional in vitro model for testing the contribution of galectins to OA pathogenesis. Thus, we evaluated if the typical dysregulation of galectin expression observed in OA cartilage is retained in chondrocytes under 3D conditions, and if Gal-1, -3, and -8 applied as an in situ-like mixture were capable of triggering functional disease markers that drive the degradation of extracellular matrix (ECM) in these 3D pellets. Finally, this study tested whether or not blocking NF-κB-dependent signaling elicited by the galectins had an impact on presence of disease markers and matrix loss.

## Methods

### Galectins

Human recombinant galectins, fluorescent galectins and rabbit polyclonal antibodies against Gal-1, -3, or -8 were prepared, purified and tested as described previously [21–23]. For details, please refer to Supplemental File 1.

### Clinical Specimens and Cell Culture

Specimens of human articular cartilage were obtained from endstage OA patients (24 female, 12 male; age range 49–84 years; Knee Society Scores: Knee Score 25–87, Functional Score 30–70; Mankin score range 6–12) during total knee replacement surgery with written informed consent and in accordance with the terms of the ethics committee of the Medical University of Vienna (EK-No. 1822/2017 and 1555/2019). Comorbidities in included patients comprised cardiovascular diseases (24 cases), obesity (15 cases), neurological pathologies (9 cases), hyperlipidemia (9 cases), nicotine abuse (6 cases), hyperuricemia (6 cases), and cancer (4 cases).

Chondrocytes were isolated from femoral condyles and tibial plateaus including OA lesions and cultured in growth medium (DMEM GlutaMAX (Gibco) supplemented with 10% fetal calf serum (Biochrom), 1% penicillin/streptomycin (Gibco), and 0.1% amphotericin B (Sigma)) in a humidified atmosphere of 5% CO_2_/95% air at 37 °C. For all assays, primary chondrocytes were used without subculturing to preserve the chondrocyte phenotype. 2D chondrocyte cultures (90% confluency) were kept overnight without serum addition and exposed to galectins combined in a standard mixture based on previous experience [21]. The NF-κB pathway was inhibited using Bay 11-7082, caffeic acid phenethyl ester (CAPE) and IKK inhibitor VII (all from Merck). Concentrations of reagents and time periods of cell treatment with reagents are listed in respective figures and their legends.

### Pellet Formation, Treatment and Analysis

5 × 10^5^ chondrocytes were seeded per 1.5 ml tubes in growth medium and centrifugated at 1000 rpm for 10 min at room temperature (RT). The pelleted cells were cultured for 2 days in growth medium, then brought into starvation medium [DMEM GlutaMAX, 1% penicillin/streptomycin mixture, 0.1% amphotericin B and 1% insulin-transferrin-selenium (Gibco)]. Following culture for 3 weeks, resulting pellets were treated with the mixture of Gal-1/-3/-8 (5 µg/ml, 1 µg/ml and 5 µg/ml, respectively) either for 48 h and for 1 week (mRNA isolation), or for 2 weeks (histological examination). An additional set of pellets was used for RT-qPCR and ELISA analyses after 2 weeks of treatment (i.e., at the time of histological evaluation), to determine the effect of CAPE. Culture medium with galectins was replaced twice a week. The pellet size was analyzed with a Nikon ECLIPSE TE2000-U microscope (×2 magnification) and NIS-Elements software (Version 4.20.03). Macroscopic pictures were taken with the ZEISS Lumar V12 equipment (×24 magnification). GAGs released into the pellet supernatant were analyzed using the DMMB assay (Supplemental File 1).

### Histology and Immunohistochemistry

Cartilage preparation, assessment of degree of degeneration using the Mankin score (MS), and immunohistochemical galectin stainings were performed as previously described [18, 21]. For details, please refer to Supplemental File 1. Pellets were processed accordingly, followed by HE, SO, and DMMB stainings, as well as immunohistochemical stainings using anti-collagen type II (mouse; Acris), anti-collagen type I (mouse; Novus Biologicals), anti-MMP-13 (mouse; R&D Systems), anti-aggrecan (mouse; Santa Cruz Biotechnology) antibodies and the set of non-cross-reactive anti-galectin antibodies. Immunohistochemical stainings were quantified using the Image J software with the plugin “Color Deconvolution” program. Of each pellet, five random pictures were recorded and quantified by splitting RGB images into defined codes (Supplemental File 1). Equal threshold settings of signal intensities were used, and the measured signal intensity was related to the average number of nuclei per image.

### RT-qPCR

Isolation of total RNA, cDNA synthesis and SYBR-green-based qPCR experiments were performed as previously described [18, 21]. Levels were calculated as relative quantities compared to controls considering amplification efficiencies and normalization to succinate dehydrogenase complex, subunit A (SDHA). A detailed checklist according to the MIQE guidelines is provided in Supplemental File 2.

### Cytochemistry Using Fluorescent Galectins

Cell suspensions of 3 × 10^5^ chondrocytes in 50 µl PBS were incubated at 4 °C for 10 min with a mixture of 2 µg/50 µl AlexaFluor647-labeled Gal-1, 4 µg/50 µl AlexaFluor488-labeled Gal-3, and 5 µg/50 µl AlexaFluor555-labeled Gal-8 in the presence or absence of cognate sugar, i.e., 0.1 M lactose. Photomicrographs were immediately taken without fixation by laser scanning microscopy at ×630 magnification (LSM700 microscope; Zeiss).

### In-Cell Western Assay

OA chondrocytes were grown in 96-well plates, exposed to the standard galectin mixture (5 µg/ml Gal-1, 1 µg/ml Gal-3, and 5 µg/ml Gal-8) fixed with methanol (-20 °C). Primary antibodies included NF-κB p65 (mouse; 1:1000; Cell Signaling) and pNF-κB p65 (Ser536; rabbit; 1:800; Cell Signaling) and secondary antibodies were donkey anti-mouse IgG IRDye 800CW (1:1000) and goat anti-rabbit IgG IRDye 680RD (1:1000). Signals were recorded using the Odyssey CLx Infrared Imaging System and Image Studio Version 5.2 (LI-COR Biosciences). For details, please refer to Supplemental File 1.

### ELISA

Cell culture supernatants were obtained from untreated pellets and pellets exposed to the galectin mixture in the presence or absence of CAPE by centrifugation and stored at −80 °C. ELISAs for pro-MMP-1, pro-MMP-13, total-MMP-3 (all from R&D Systems), Gal-1 (R&D Systems), Gal-3 (R&D Systems), and Gal-8 (Cloud-Clone Corp.) were performed with culture supernatants following the manufacturers’ protocols. ELISA standard curve ranges were 0.156–10 ng/ml (pro-MMP-1, total-MMP-3, Gal-3, Gal-8), 0.313–20 ng/ml (Gal-1), and 78–5000 pg/ml (pro-MMP-13).

### Cell Viability Assays

Cell viability of chondrocytes and pellets was determined with the EZ4U assay (Biomedica) and the CyQUANT LDH Cytotoxicity Assay (Thermo Fisher Scientific), respectively, according to the manufacturers’ instructions. For details, please refer to Supplemental File 1.

### Statistics

All experiments were repeated independently with primary chondrocytes from different OA patients. The number of biological replicates (i.e., different patients) is given in the respective figure legends. Technical replicates of each biological replicate were averaged and the resulting mean values were used for statistics. Statistical analyses were performed using SPSS 25.0. Normal distribution of the data was analyzed using the Shapiro–Wilk test. Statistical significance of normally distributed data was delineated using paired *t*-test or ANOVA with Dunnett’s post hoc test. For non-normally distributed data, the Wilcoxon test or Friedman test with pairwise comparison was used. *p*-Values < 0.05 were considered significant.

## Results

### Phenotypic, Immunohistochemical and Transcriptional Characterization of OA Chondrocyte Pellet Cultures

**Fig. 1:**
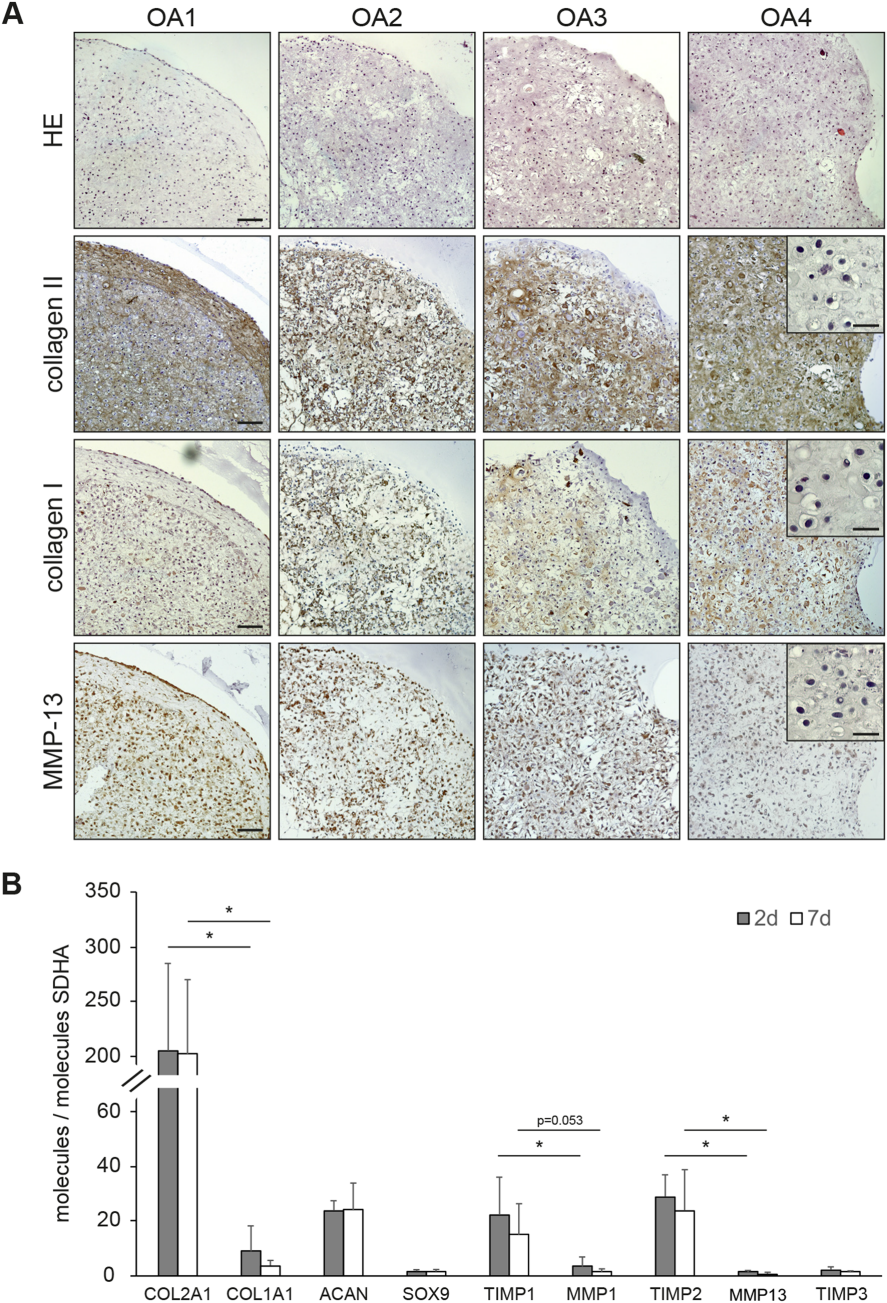
3D pellet cultures maintain features of the OA chondrocyte phenotype. **a** Consecutive histological sections of OA chondrocyte pellets from four patients (OA1-4) were stained with HE or immunohistochemically stained for collagen type II, collagen type I, or MMP-13 (3 technical replicates). Representative negative controls for each immunohistochemical staining, prepared by omission of the incubation step with primary antibody from processing, were added as insets to OA4. Scale bars: 100 µm (main images, exemplarily depicted in OA1), 20 µm (insets). **b** mRNA expression levels of cartilage marker genes are given as relative copy numbers with respect to the abundance of SDHA. cDNA of OA chondrocyte pellets established from specimens of four donors were analyzed using qPCR (2 technical replicates) after 2 and 7 days of culture; mean values and standard deviations are given. Significant differences between the levels of selected mRNA targets are indicated with asterisks (*p < 0.05, *n* = 4, paired one-sided *t*-test)

After 5 weeks of culture, chondrocyte pellets from four OA patients were histologically processed. HE-stained sections revealed cells of round morphology in lacunae, surrounded by considerable amounts of ECM (Fig. 1a). Immunohistochemical analysis revealed a pronounced staining of the ECM for collagen type II, the main collagenous component of native articular cartilage (Fig. 1b). Positivity of the ECM for collagen type I was also found (Fig. 1a). Despite some interindividual variability regarding staining intensities between the donors, collagen type II was far more abundant than collagen type I in each of the four independent cell populations, as it is expected for articular chondrocytes. In addition, the ECM was positive in 2 cases for glycosaminoglycans (SO and DMMB staining) and in 3 cases for aggrecan (Supplementary File 3). Immunohistochemistry further developed strong signals for the presence of MMP-13, a major collagenase with a key role in cartilage degeneration, in the cytoplasm of chondrocytes (Fig. 1a, bottom). This staining demonstrates the intrinsic ability of the OA-derived cells in pellets to produce this typical catabolic enzyme (Fig. 1a).

Additional characterization of the pellet cultures was performed at the level of mRNA expression (Fig. 1b). In line with the immunohistochemical findings, transcripts of COL2A1 were significantly more abundant than those of COL1A1, resulting in COL2A1/COL1A1 ratios of 61 ± 25 (*n* = 4 OA patients). The mRNA levels of the aggrecan-encoding gene (ACAN) as well as that of the chondrocyte differentiation factor SOX9 were detectable at levels exceeding that of the reference gene SDHA. The extent of expression of the ‘tissue inhibitors of metalloproteinases’ TIMP-1 and TIMP-2 (but not of TIMP-3) was significantly higher than that of MMP-1 and MMP-13, respectively, resulting in a TIMP-1/MMP-1 ratio of 24 ± 36 and a TIMP-2/MMP-13 ratio of 50 ± 58.

Taken together, these data demonstrate (i) the maintenance of the chondrocyte phenotype in 3D pellet cultures over several weeks of culture, (ii) the accumulation of cartilage-like ECM and (iii) the expression of catabolic enzymes, thereby suggesting the suitability of this model for the functional investigation of galectin activity on ECM degradation in vitro.

### Characterization of Galectin Expression and Secretion in OA Chondrocyte Pellet Cultures

**Fig. 2:**
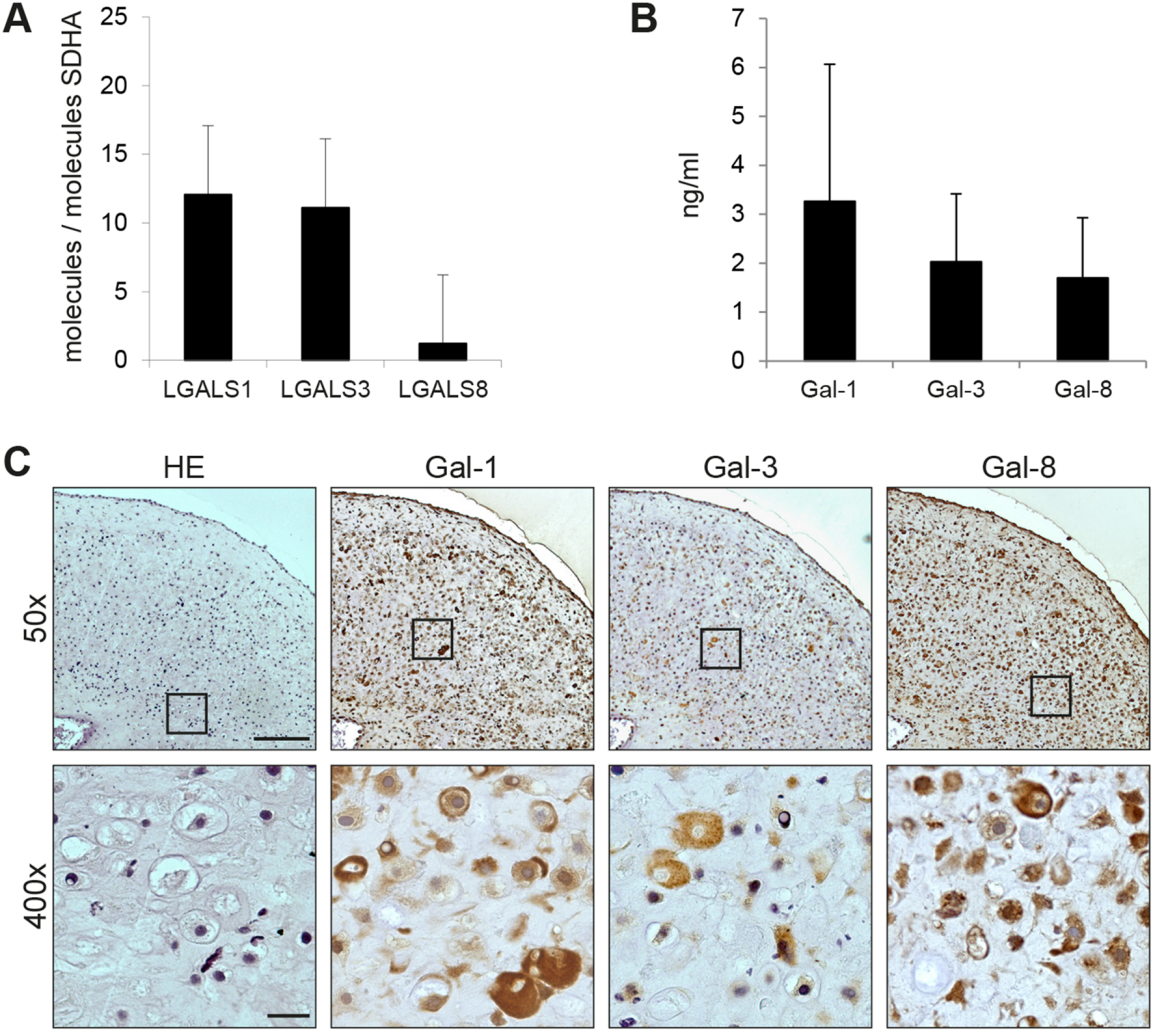
OA chondrocyte pellets express and secrete galectins. **a** mRNA expression levels of LGALS1, LGAL3 and LGAL8 were calculated as relative copy numbers with respect to the abundance of SDHA. cDNA of OA chondrocyte pellets obtained by culturing material from six donors was analyzed using qPCR after 7 days of culture (2 technical replicates); mean values and standard deviations are given. **b** Bar chart shows levels of secreted galectins (measured using ELISA) in the supernatant of OA chondrocyte pellets from four donors as mean values and standard deviations (no technical replicates). **c** Consecutive histological sections of OA chondrocyte pellets from three donors were stained with HE and immunohistochemically stained for Gal-1, -3 or -8 (3 technical replicates). Shown is a series of stainings of pellets from one representative patient at ×50 magnification (upper row). Areas marked with rectangles are additionally presented at ×400 magnification (bottom row). Scale bars (exemplarily depicted in HE images): 200 µm (×50), 20 µm (×400)

We next proceeded to examine the expression of Gal-1, -3, and -8 in the OA chondrocyte pellet model at the level of transcription and of secretion. Quantification of their mRNA levels disclosed high signal intensities, exceeding that of the reference gene SDHA (Fig. 2a). Since secretion of effector proteins is a prerequisite for triggering cell signaling via cell surface receptors, we next analyzed culture supernatants for presence of these galectins. As shown in Fig. 2b, presence of galectins in the culture medium of pellets was detected in each case. Thus, cells in the 3D culture system can export galectins after their production on free ribosomes. This initial location without common entry to the endoplasmic reticulum is visualized by the predominant cytoplasmic localization of the galectins throughout cross sections of histological specimens (Fig. 2c).

In order to unravel similarities between the 3D culture model and the physiologic setting in vivo, clinical specimens were processed in parallel under identical conditions, using serial sections for assessing galectin presence and their distribution as network. As illustrated in Supplemental File 4(A), staining profiles for the three galectins monitored simultaneously in OA cartilage (with the typical upregulation in relation to an increase in the MS) closely resembled the pattern in the sections of pellet cultures. The sizes of the Spearman correlation coefficients and of associated significance levels confirmed the expectation for a positive association among the galectins [Supplemental File 4(B) and (C)]. Pellet cultures obviously retained the typical upregulation of galectins, previously seen in clinical specimens and in 2D OA chondrocyte cultures [19–21]. In order to establish pellet cultures as tools for studying therapeutic means in vitro to interfere with disease onset and progression, we further pursued work with pellets to answer the pertinent question whether galectins will promote typical characteristics of disease manifestation in these models. Thus, we aimed to evaluate the impact of a mixture of Gal-1, -3, and -8 (i.e., Gal-1/-3/-8) on functional ECM breakdown in vitro.

### Galectins as Inducers of Matrix Degradation and Functional Disease Markers Via NF-κB and Effect of CAPE as Inhibitor

**Fig. 3:**
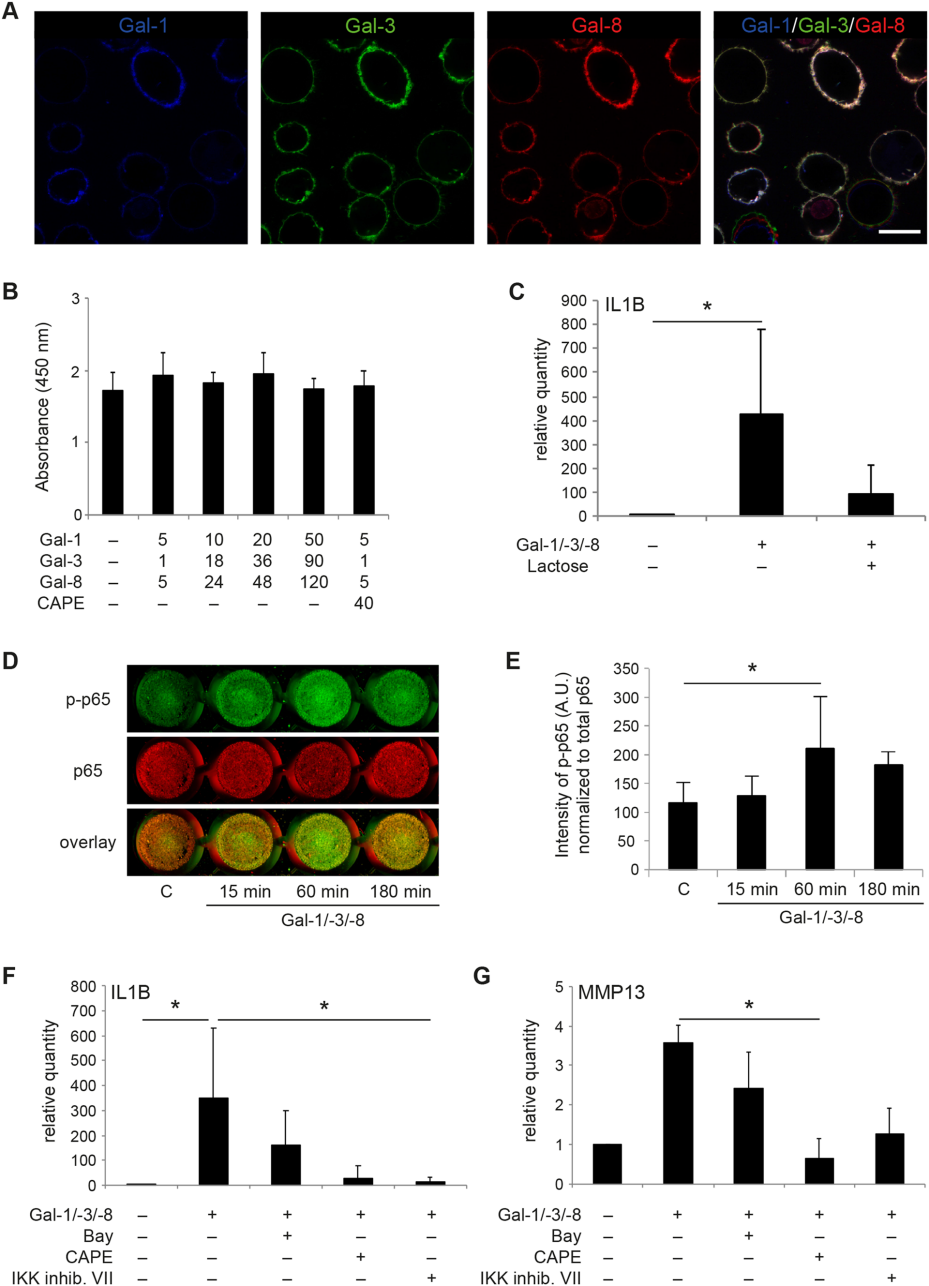
Gal-1/-3/-8 activates the NF-κB pathway in OA chondrocytes. **a** Localization of binding sites for fluorescent Gal-1, Gal-3, and Gal-8 on isolated chondrocytes in vitro. Cultured chondrocytes from three donors in passage 0 were trypsinized and resuspended prior to incubation with the three dye-galectin conjugates Gal-1-AlexaFluor647 (blue), Gal-3-AlexaFluor488 (green) and Gal-8-AlexaFluor555 (red) at 4 °C for 10 min. Then, cells were washed and analyzed using laser scanning microscopy. Shown is a series of images of cells from one representative patient. Scale bar: 20 μm. **b** Viability assay of OA chondrocytes from three donors was performed after 24 h treatment with various concentrations of Gal-1/-3/-8 in the presence or absence of CAPE (4 technical replicates). **c** OA chondrocytes from three donors were treated with the galectin mixture Gal-1/-3/-8 (5/1/5 µg/ml) in the absence or presence of 0.2 M lactose for 24 h. Relative quantities of IL1B mRNA levels (normalized to SDHA) were evaluated using RT-qPCR with respect to untreated control cells set to 1 (2 technical replicates). Significant differences between groups are indicated with asterisks (*p < 0.05, *n* = 3, Friedman test). **d**, **e** OA chondrocytes from seven patients were exposed to the galectin mixture Gal-1/-3/-8 (5/1/5 µg/ml) over time and NF-κB activation was measured by In-Cell Western (2 technical replicates). **d** A scan of cells from one representative patient is shown. **e** Signal intensities of phosphorylated p65 were normalized to total p65 and shown as mean values and standard deviations (*n* = 7). Significant differences to untreated control cells set to 1 are indicated with asterisks (*p < 0.05, *n* = 7, ANOVA with Dunnett’s post hoc test). **f**, **g** Bar charts show relative quantities (mean values and standard deviations, *n* = 5) of mRNA levels of IL1B (**f**) and MMP-13 (**g**), measured using RT-qPCR (2 technical replicates) with untreated control values set to 1. The galectin mixture Gal-1/-3/-8 (5/1/5 µg/ml) and a distinct concentration of each of the NF-κB inhibitors (4 µM Bay 11-7082, 40 µM CAPE, or 4 µM IKK inhibitor VII) were present in the medium for 24 h (*n* = 5 patients). Significant differences between groups are indicated with asterisks (*p < 0.05, *n* = 5, Friedman test)

Setting an internal standard for these functional assays in pellets, we first proceeded with experiments in 2D-cultured OA chondrocytes. The obtained results demonstrated the ability of Gal-1, -3, -and -8 to simultaneously bind to OA chondrocytes when applied as mixture to mimic the in vivo condition (Fig. 3a). In addition, the data, here obtained by using 3-color fluorescence microscopy, confirmed previous results on galectin-surface counterreceptor pairing [19–21]. Presence of the cognate sugar lactose completely abolished the binding of the three galectins, excluding a carbohydrate-independent binding process (not shown). Neither the mixture of galectins nor the presence of CAPE affected chondrocyte viability (Fig. 3b). Whereas IL-1β-specific mRNA levels were upregulated by Gal-1/-3/-8, the additional presence of lactose alleviated this induction (Fig. 3c), highlighting glycan binding of the galectins as a prerequisite for triggering an effect. Lactose (without Gal-1/-3/-8) did not significantly modulate IL1B mRNA levels in this experimental setting (not shown). Intracellularly, the increase of phosphorylated p65 as sensor of galectin-dependent NF-κB activation underscored the activity of the galectin mixture at this point (Fig. 3d and e), again confirming and extending previous observations made in assays with separately tested galectins [19–21]. The involvement of NF-κB signaling prompted us to select an appropriate inhibitor in the attempt to attenuate the expression of functional disease markers. Among the three compounds tested, CAPE actively reduced the mRNA levels of IL1B (Fig. 3f) and MMP-13 (Fig. 3g), which were selected as representative indicators for the disease-associated inflammatory and degradative processes.

**Fig. 4:**
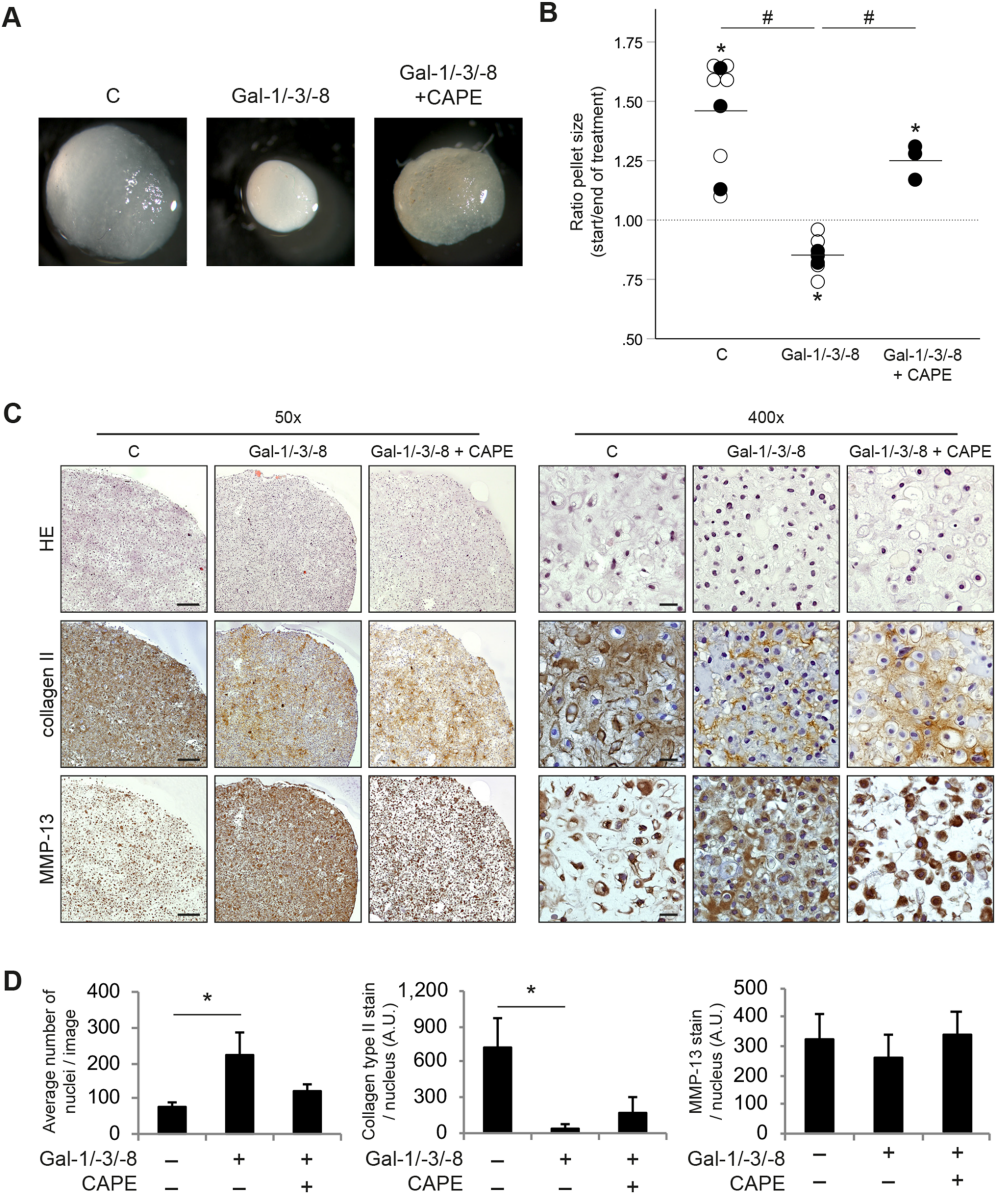
Extracellular matrix degeneration in OA chondrocytes pellets after treatment with the galectin mixture Gal-1/-3/-8 and CAPE. **a** OA chondrocyte pellets from five donors were cultured for 3 weeks followed by 2 weeks of treatment with Gal-1/-3/-8 (5/1/5 µg/ml) in the absence or presence of CAPE (40 µM). Untreated pellets were used as control (**c**). Shown are macroscopic pictures of OA chondrocyte pellets from one representative patient. **b** Shown is the ratio of the pellet size between start (day 21) and end of treatment (day 35). Pellets were treated with Gal-1/-3/-8 (*n* = 7 patients), or with Gal-1/-3/-8 + CAPE (*n* = 3 patients). Control pellets were left untreated (*n* = 9 patients). Each circle represents one patient. Full circles illustrate the three cell populations that were cultured under all three conditions. Lines indicate the mean values of each group. The dashed line marks the ratio of 1, indicating a hypothetical stable pellet size during treatment. Asterisks indicate statistical significant difference of a group compared to a value of 1 (“stable size”; two-sided *t*-test, *p* < 0.05). Hash signs indicate significant differences between groups (ANOVA with Tukey post hoc test, *p* < 0.05). **c** Consecutive histological sections of OA chondrocyte pellets from three donors were stained with HE, or with anti-collagen type II and anti-MMP-13 antibodies (3 technical replicates; left panel, ×50 magnification; right panel, ×400 magnification). Shown is a series of stainings of pellets from one representative patient. Scale bars (exemplarily depicted in images of untreated control pellets): 200 µm (×50), 20 µm (×400). **d **Bar charts show the average number of nuclei (left graph), signal intensity of collagen type II-staining normalized to the number of nuclei (middle graph), and signal intensity of MMP-13 staining normalized to the number of nuclei (right graph) across three patients (5 technical replicates). Significant differences between the groups are indicated with asterisks (*p < 0.05, *n* = 3, Friedman test)

With this experience in hand, we proceeded to expose pellet cultures to the galectin mixture in the absence or presence of CAPE. Shown exemplarily as photomicrographs (Fig. 4a) and as dot plot after quantitative analysis (Fig. 4b), the galectin mixture was effective to reduce the pellet size during the course of treatment, while the presence of CAPE ameliorated this status. HE-stained histological sections of the treated pellets showed no obvious signs of necrosis or apoptosis that might explain the reduction of pellet sizes after treatment with Gal-1/-3/-8 (Fig. 4c). Additional analyses of LDH activity in supernatants of pellets support the absence of cytotoxic effects of a Gal-1/-3/-8 treatment in pellet cultures (not shown). As reference, untreated control pellets were characterized by a small number of chondrocytes per field of view and highly abundant ECM that was strongly immunopositive for collagen type II (Fig. 4c). Treatment with the galectin mixture, however, resulted in a significantly increased cell density per field of view (Fig. 4d), accompanied by a significantly reduced intensity of staining of the ECM for collagen type II (Fig. 4c and d). Of note, the additional treatment of pellets with CAPE alleviated these effects of Gal-1/-3/-8, resulting in cell density and collagen type II-staining that was comparable to untreated pellets (*p* > 0.05; Fig. 4c and d). Immunohistochemical staining for MMP-13 resulted in an apparently increased staining of chondrocytes in the Gal-1/-3/-8-treated pellets per field of view (Fig. 4c). Since this staining concerned the chondrocytes and not the ECM, normalization to the number of cells was mandatory. When done, the obtained data made clear that neither Gal-1/-3/-8 nor CAPE had an effect on the presence of MMP-13 in the chondrocytes within the pellets (Fig. 4d). In those cases where the pellet ECM was positive for GAGs and aggrecan, treatment with Gal-1/-3/-8 reduced this positivity [Supplementary File 6(A)]. Analysis of pellet supernatants of 13 OA patients indicated levels of GAGs between 8 and 53 µg/ml, which were significantly reduced by Gal-1/-3/-8 [*p* < 0.05; Supplementary File 6(B)]. Addition of CAPE was not able to recover this GAG loss (*p* > 0.05; *n* = 9; not shown).

**Fig. 5:**
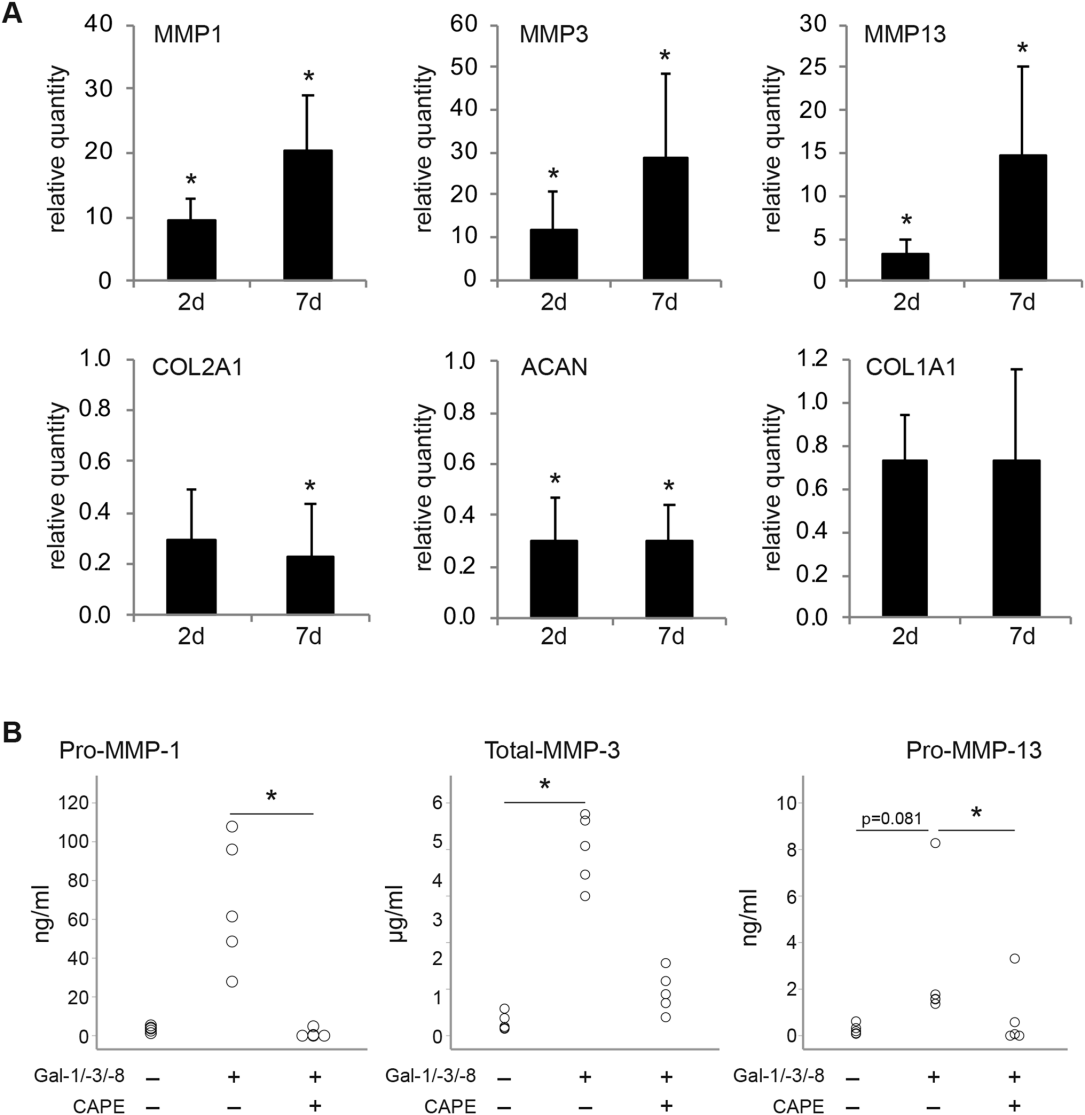
OA markers are modulated in OA chondrocyte pellets after treatment with the mixture of Gal-1/-3/-8 (5/1/5 µg/ml). **a** Bar charts show the relative quantities of mRNA levels of MMP-1, MMP-3, MMP-13, COL2A1, ACAN, and COL1A1 (measured using RT-qPCR; 2 technical replicates) in pellets treated with the galectin mixture for either 2 (2 days; *n* = 4 patients) or 7 days (7 days; *n* = 5 patients). Significant differences to experimental data of untreated control pellets set to 1 (not shown) are indicated with asterisks (*p < 0.05, *n* = 4 or 5, paired *t*-test). Paired *t*-tests of MMP-1, MMP-3, MMP-13, COL2A1 and ACAN mRNA data were performed by one-sided testing, since previous studies had proven one-sided regulation of these genes after galectin treatment [19–21]. COL1A1 data were analyzed by two-sided testing. **b** After 2 weeks of treatment (i.e., at the time of histological evaluation), an additional set of pellets (*n* = 5 patients) was used for ELISA (no technical replicates) analyses, to determine the effect of CAPE. Dot plots show the concentrations of pro-MMP-1, total-MMP-3, and pro-MMP-13 in the supernatants of OA chondrocyte pellets (secreted within 48 h), treated with the galectin mixture Gal-1/-3/-8 (5/1/5 µg/ml) in the presence or absence of CAPE (40 µM). Significant differences between groups are indicated with asterisks (*p < 0.05, *n* = 5, Friedman test)

To identify the molecular triggers that are responsible for the observed ECM breakdown induced by the galectin mixture, RT-qPCR analyses and ELISAs were performed in chondrocyte pellets that were treated with the galectin mixture for 2 or 7 days. Figure 5a shows that the mRNA levels of MMP-1, MMP-3, and MMP-13 were significantly upregulated at both time points, whereas transcripts of COL2A1 and ACAN were markedly downregulated. The COL1A1 mRNA level, in contrast, was not significantly affected. At the end of the culture period (i.e., after 2 weeks of treatment, when histology was performed), protein levels of secreted MMPs were strongly induced by Gal-1/-3/-8, i.e., from 3.4 ± 1.5 to 68.4 ± 33.2 ng/ml pro-MMP-1 (*p* > 0.05), from 0.3 ± 0.2 to 4.0 ± 0.8 µg/ml total-MMP-3 (*p* < 0.05), and from 0.3 ± 0.2 to 2.9 ± 3.0 ng/ml pro-MMP-13 (*p* = 0.81). The presence of CAPE evoked the significant reduction to basal levels in case of pro-MMP-1 (1.0 ± 2.1 ng/ml) and pro-MMP-13 (0.8 ± 1.4 ng/ml). Albeit not statistically significant, Gal-1/-3/-8-induced total-MMP-3 levels were alleviated by CAPE in all samples (0.9 ± 0.4 µg/ml).

## Discussion

A key challenge for OA research is to define in molecular terms the steps from elicitors to effectors, and then to the progressive degradation of articular cartilage including the detrimental crosstalk to underlying subchondral bone [2, 24]. Thus, availability of reliable in vitro disease models that intend to simulate the degenerative phenotype (e.g., by applying genome editing, as recently suggested for intervertebral disk degeneration [25]) is required. Ideally, such in vitro models should morphologically and functionally resemble the in vivo situation, along with retaining the capacity of the cultured OA chondrocytes to respond to drivers of pathogenesis by increasing functional biomarkers. As illustrated by the presented results, 3D pellet cultures of OA chondrocytes appear to meet these prerequisites for the measured characteristics. Most importantly, OA chondrocyte pellets facilitated the evaluation and quantification of the degradation of collagen type II-rich ECM, together with the assessment of associated functional markers at the mRNA and protein levels, in response to galectins in vitro.

Of conceptual significance, functional activity was determined in all assays with a mixture of the three galectins that share capacity for upregulation of disease markers. The possibility for teamworking among galectins in OA had been suggested by our work, and respective initial testing had been performed previously [21]. In general overview, galectins had first been detected and analyzed for functionality in a strictly separated manner. In few cases, evidence for a cooperation among galectins had been detected, first for proto-type Gal-1 and chimera-type Gal-3 in human neuroblastoma cells [26] and for proto-type Gal-1, -2, and -7 in human activated T cells to activate different caspase profiles/cyclin B_1_ expression [27], then for Gal-1 and -3 in human activated neutrophils to initiate phosphatidylserine exposure [28] as well as for Gal-1 and -8 to promote plasma cell formation [29] and to enhance antigen-specific stimulation of murine naïve peripheral CD4^+^ T cells [30]. Just as approaching the histological architecture in vivo by proceeding from 2D to 3D culture, the components of the galectin network should no longer be tested separately, but combined in mixtures to purposefully mimic the situation in vivo. Moreover, this co-incubation would ensure to include protein species into the functional analysis that are known to form in such mixtures, i.e., heterodimers [31].

Having revealed the suitability of OA chondrocyte pellets for studies on the role of galectins in disease onset and progression, we further tested the hypothesis that OA chondrocyte pellets can serve as an in vitro model to trace a pharmacological attenuation of OA features. After comparative testing of three inhibitors in vitro, CAPE was selected as test substance for this proof-of-concept study. CAPE, which had previously been described as an effective inhibitor of NF-κB-dependent expression of Gal-7 in breast cancer cells [32], was applied to Gal-1/-3/-8-treated OA chondrocyte pellets and, in this model, led to a strong reduction of signs of disease progression, i.e., ECM breakdown and biomarker expression. Mechanistically, these results underscored the capacity of the tested galectin mixture to cause tissue degradation via NF-κB activation. Since stimulation of this pathway by galectins has been implicated in processes associated with fibrosis, i.e., in increased production of the chemokines CXCL1 and CCL2 by rat pancreatic stellate cells (via Gal-1) [33] and in enhanced interleukin-8 secretion by colonic lamina propria fibroblasts (via Gal-3) [34], galectin-triggered NF-κB signaling deserves attention even beyond OA.

Of note, NF-κB can also modify the responsiveness of cells to galectins. Toward this end, NF-κB has been postulated to promote the extent of functional pairing of Gal-1 on activated human T (L7) cells to effectively induce apoptosis by increasing gene expression, in cooperation with Sp1, of its counterreceptor CD7 [35]. This glycoprotein is required for Gal-1-mediated T cell death in vitro and in vivo [36]. Consequently, further work in OA chondrocytes should aim at the identification of the binding partner(s) of the galectins that trigger the first steps of the post-binding signaling cascade toward NF-κB activation. The case of carcinoma growth regulation by Gal-1, which is started by lattice formation with α_5_β_1_-integrin that is switched on/off by regulating extent of α2,6-sialylation of the glycoprotein’s *N*-glycans, offers a precedent, in which expression of galectin and counterreceptor are intimately orchestrated [37]. Of note, on the cell surface, a galectin can not only serve as crosslinker for lattice formation with its counterreceptor [38, 39], but it can also act as molecular glue associating more than one binding partner in a ternary or even higher-order complex by protein-glycan/protein recognition [40].

Equally important, pellet cultures can likewise be instrumental to address the pertinent question on the nature of the elicitor(s) that induce the concerted increase of galectin expression. Candidates among these elicitors are Runt proteins and hypoxia-inducible factors [41], which have been demonstrated to affect Gal-1 and/or Gal-3 gene transcription [42, 43]. Elicitors that act further upstream in OA pathogenesis are not yet known. Of interest in this context, butyrate is an example for a small molecule enhancer of Gal-1 expression in human cancer cells [44]. Activation with anti-CD3/CD28 or reconstitution of the tumor suppressor (p16^INK4a^) status document the presence of efficient routes for upregulating Gal-1 expression in CD4^+^CD25^+^ regulatory T cells, along with increased counterreceptor presence [45].

Finally, it is anticipated to apply the described pellet model to evaluate the efficacy of therapeutic agents that interfere with galectin activity. In this context, 3D pellets are illustrated to provide a valuable asset to study the functional process of ECM degradation under controlled conditions in vitro.

## Conclusion

Application of OA chondrocyte pellets revealed that a mixture of Gal-1, -3, and -8 triggered ECM breakdown along with the upregulation of functional disease markers, underscoring the deleterious role of the combined presence of these galectins in the pathobiology of OA. Providing therapeutic perspective, the presence of CAPE alleviated the process of ECM degradation via inhibition of NF-κB signaling. This study therefore proposes the use of this model as a practical in vitro disease model to study the (galectin-induced) degradation of cartilage-like ECM and the inhibition of this process using pharmacological agents. Future studies should integrate this model as a tool in experiments to identify the counterreceptors and the elicitors of galectins, and to discover strategies that might suppress the ECM-degrading potential of galectins in OA.

## Electronic supplementary material

Below is the link to the electronic supplementary material.

Electronic supplementary material 1 (PDF 81 kb)

Electronic supplementary material 2 (PDF 74 kb)

Supplemental file 3 Consecutive histological sections of OA chondrocyte pellets from four patients (OA1-4) were stained with SO, DMMB, or immunohistochemically stained for aggrecan (3 technical replicates). Arrowheads indicate examples of positivity. Representative negative control for immunohistochemical staining is added as inset to OA4. Scale bars: 50 µm (main images, exemplarily depicted in OA4), 20 µm (inset). (PDF 778 kb)

Supplemental file 4 Cartilage degeneration is accompanied by the increase of chondrocyte positivity for Gal-1, -3 and -8 measured immunohistochemically. (a) Consecutive sections of articular knee cartilage from 10 OA patients were stained with Safranin O (SO) and immunohistochemically stained for Gal-1, Gal-3, or Gal-8 (3 technical replicates). Shown is a series of stainings of pellets from one representative patient. MS 2 = Mankin score 2; MS 10 = Mankin score 10. Scale bars: 200 µm (SO staining), 20 µm (galectin stainings). (b) Correlation analyses of Gal-1, Gal-3 and Gal-8 shown as pairwise scatter plots for 70 measurements (3 technical replicates). (c) Statistical outcome of correlation analyses between the positivities for the three galectins including Spearman correlation coefficients and p-values. (PDF 426 kb)

Supplemental file 5 Comparison of the sets of proteoglycan-related regulated by Gal-1, Gal-3 or Gal-8S as determined by microarray analysis. The ratios between mRNA levels of genes in Gal-1, Gal-3 or Gal-8S-treated versus untreated chondrocytes as well as p-values, corrected for multiple hypothesis testing by the Benjamini–Hochberg method, are given. Data reproduced from GEO (accession numbers: Gal-1: GSE68760, Gal-3: GSE85254, Gal-8S: GSE131822). (PDF 63 kb)

Supplemental file 6 (a) OA chondrocyte pellets from four donors were cultured for three weeks followed by two weeks of treatment with or without Gal-1/-3/-8 (5/1/5 µg/ml). Consecutive histological sections of OA chondrocyte pellets were stained with SO, DMMB, or with anti-aggrecan antibodies (3 technical replicates; left panel, 50x magnification; right panel, 400x magnification). Shown is a series of stainings of pellets from one representative patient (i.e., OA3 in Supplementary file 4). Scale bars (exemplarily depicted in images of untreated control pellets): 200 µm (50x), 50 µm (400x). (b) OA chondrocyte pellets from 13 donors were cultured for three weeks followed by two weeks of treatment with or without Gal-1/-3/-8 (5/1/5 µg/ml). Supernatants of pellets were evaluated for the presence of GAGs (µg/ml) using the DMMB method. Significant differences between groups are indicated with asterisk (*p<0.05, n=13, Wilcoxon test). (PDF 851 kb)
