# Supplementary material for: The Dysregulated Galectin Network Activates NF-κB to Induce Disease Markers and Matrix Degeneration in 3D Pellet Cultures of Osteoarthritic Chondrocytes
Source: Calcif Tissue Int. 2020 Nov 13;108(3):377–90. doi: 10.1007/s00223-020-00774-4 (PMC7881967; doi:10.1007/s00223-020-00774-4)
Supplement: Supplementary file 1 — Electronic supplementary material 1 (PDF 81 kb) [file 223_2020_774_MOESM1_ESM.pdf]

Supplemental file 1. Background methods.

Galectins

Human galectins were obtained by recombinant production and purified by affinity chromatography on lactose-presenting resin as crucial step, using 1D and 2D gel electrophoresis, mass spectrometry and gel filtration to ascertain purity and haemagglutination of trypsin-treated, glutaraldehyde-fixed rabbit erythrocytes to document activity [25, 26]. Their labeling by commercial fluorescent dyes was performed under activity-preserving conditions, and the fluorescent products were analyzed for extent of conjugation and for maintained activity of glycan binding by cytofluorimetry and galectin histochemistry [27]. Galectins were routinely stabilized by letting reactive sulfhydryl groups of Cys residues become modified by iodoacetamide. The preparations of rabbit polyclonal antibodies against Gal-1, -3 or -8 obtained as immunoglobulin G (IgG) fraction of serum by affinity chromatography over resin-immobilized protein A were rigorously checked by systematic ELISAs for cross-reactivity against other human galectins, and cross-reactive material was removed chromatographically by retention on affinity resin presenting covalently attached galectins as ascertained by performing another round of ELISAs [28].

Histology and immunohistochemistry

Deparaffinized consecutive cartilage tissue sections were incubated with galectin-type-specific polyclonal rabbit anti-galectin IgG preparations against either human Gal-1, -3 or -8 and a second-step reagent. Staining was developed using horseradish peroxidase-containing kit reagent (VECTASTAIN Elite ABC Kit, Vector Labs) as well as 3,3′-diaminobenzidine tetrahydrochloride hydrate (Fluka) and H_2O_2 as dye-generating substrates. Sections were

counterstained using Mayer's hemalum solution (Merck). Cell positivity for each target protein in the regions of interest was independently assessed by two experienced observers.

Of each pellet, five random pictures were recorded and quantified by splitting RGB images into the following codes (COL2A1: R0: 0.3186449, G0: 0.5210289, B0: 0.7918297, R1: 0.5184601, G1: 0.7035719, B1: 0.48598942, R2: 0.60740095, G2: 0.5168006, B2: 0.6033086; MMP-13: R0: 0.40692636, G0: 0.56900454, B0: 0.7145941, R1: 0.6167847, G1: 0.6566699, B1: 0.43400604, R2: 0.92310935, G2: 0.33087113, B2: 0.19594249).

DMMB staining

Pellets were embedded in paraffin according to standard procedures. Following deparaffinization, sections (2.5 µm) were stained with 1% DMMB solution for 15 min. Then, sections were washed twice with 70% ethanol, once with 96% ethanol and once with isopropanol. Finally, sections were incubated with xylol for 10 min and mounted for microscopy.

GAG quantification

Samples of pellet culture medium collected after two weeks of treatment were analyzed for sulfated GAG content using DMMB assay. Standards contained up to 50 µg/ml chondroitin sulfate sodium salt from shark cartilage (Sigma Aldrich) and were prepared in pellet culture medium. For the assay, 20 µl sample or standard were mixed on a 96-well microtiterplate with 200 µl DMMB reagent. The absorbance was measured immediately at 520 nm with a TECAN Spark 10 M microplate reader.

Cell viability assays

Cell viability of chondrocytes was profiled by testing increasing galectin and CAPE concentrations with the EZ4U assay system (Biomedica). In brief, 5 x 10^3 cells per well were cultured in a 96-well plate. At 80% confluency, the treatment was performed in full medium for 24 h at 37°C. Assay solutions were removed, and cells were incubated with mixed EZ4U kit substrates for 4 h at 37°C. Absorbance was measured at 450 nm with FLUOstar optima microplate reader (BMG Labtech).

Lactate dehydrogenase (LDH) activity was determined by a commercial kit following the instructions of the manufacturer (CyQUANT™ LDH Cytotoxicity Assay Kit; Thermo Fisher Scientific). In brief, OA pellets were cultured for three weeks, followed by two weeks of culture in the absence or presence of the galectin mixture (control; Gal-1/-3/-8 (5/1/5 µg/ml, without additive or with CAPE (40µM)). Pellets consisting of 4 x 10^5 cells were lysed and the suspension was incubated after adding reaction mixture for signal generation. After adding stop solution, absorbance was measured at 490 nm and 680 nm with a TECAN Spark 10m instrument.

In-Cell Western (ICW) assay

OA chondrocytes were grown in 96-well plates until they reached 100% confluency. Cells in growth medium were exposed to the standard galectin mixture (5 µg/ml Gal-1, 1 µg/ml Gal-3, and 5 µg/ml Gal-8) for indicated periods of time. Afterwards, cells were washed once with phosphate-buffered saline (PBS) and fixed with methanol (-20°C) for 10 min at RT, followed by removing methanol and thorough rinsing with PBS. Then, sites for unspecific protein binding on cells were saturated with blocking buffer (LI-COR Odyssey blocking buffer) over a period of at least 90 min under moderate shaking at RT, followed by overnight incubation at 4°C with solutions containing primary antibodies diluted in blocking buffer (NF-κB p65, mouse

at 1:1000, Cell Signaling; pNF-κB p65 (Ser536), rabbit at 1:800, Cell Signaling). After 12 h, cells were washed five times with PBS/Tween 20 (0.1%) to remove reagents, followed by stepwise incubation steps with secondary antibody-containing solution (donkey anti-mouse IgG IRDye^{TM} 800CW, at 1:1,000; then goat anti-rabbit IgG IRDye^{TM} 680RD at 1:1,000; LI-COR Biosciences) dissolved in blocking buffer containing Tween 20 (0.2%) for 1 h at RT. After five washes with PBS/Tween 20 (0.1%), each plate was dried and scanned with Odyssey CLx Infrared Imaging System (LI-COR Biosciences). Finally, the ratio between phosphorylated and total p65 was determined using the software provided by the manufacturer (Image Studio Version 5.2).
