# Supplementary material for: The Dysregulated Galectin Network Activates NF-κB to Induce Disease Markers and Matrix Degeneration in 3D Pellet Cultures of Osteoarthritic Chondrocytes
Source: Calcif Tissue Int. 2020 Nov 13;108(3):377–90. doi: 10.1007/s00223-020-00774-4 (PMC7881967; doi:10.1007/s00223-020-00774-4)
Supplement: Supplementary file 2 — Electronic supplementary material 2 (PDF 74 kb) [file 223_2020_774_MOESM2_ESM.pdf]

Supplemental file 2. RT-qPCR checklist providing relevant information on the used protocols.

Sample/Template	details
Source	Cultured human osteoarthritic chondrocytes, isolated enzymatically from clinical specimens of patients undergoing total knee replacement surgery.
Method of preservation	No preservation; Total RNA was extracted directly from PBS washed micromass pellets or cell monolayers.
Storage time (if appropriate)	RNA was stored at -80°C and reverse transcribed into cDNA within one month. cDNA was stored at -80°C and analyzed within 1 year.
Handling	----
Extraction method	Column based extraction method (innuPREP RNA Mini Kit (Analytik Jena, Germany)).
RNA: DNA-free	All primer pairs used are intron-spanning primers. Each RNA sample was examined for purity and quantity using the NanoDrop 1000. 'Minus RT controls' were prepared from random RNA samples.
Concentration	Each RNA sample was examined for purity and quantity using the NanoDrop 1000.
RNA: integrity	The process used for RNA isolation is periodically verified by checking the RNA integrity of samples using microfluidics (Bioanalyzer).
Inhibition-free	Not all samples were tested for the absence of inhibitors. However, a representative set of samples was used to control for the absence of inhibitors using dilution series of target genes.
**Assay optimisation/validation**	
Accession numbers	
IL1B	NM_000576
COL2A1	NM_001844
COL1A1	NM_000088
ACAN	NM_001135
SOX9	NM_000346
TIMP1	NM_003254
TIMP2	NM_003255
TIMP3	NM_000362
SDHA	NM_004168
MMP1	NM_002421
MMP3	NM_002422
MMP13	NM_002427
LGALS1	NM_002305
LGALS3	NM_002306
LGALS8	NM_006499
Amplicon details	
IL1B	132 bp
COL2A1	377 bp
COL1A1	179 bp
ACAN	194 bp
SOX9	180 bp
TIMP1	195 bp
TIMP2	153bp
TIMP3	164 bp
SDHA	86 bp
MMP1	144 bp
MMP3	184 bp
MMP13	77 bp
LGALS1	125 bp
LGALS3	165 bp
LGALS8	91 bp
Primer sequence	
	**forward**
IL1B	CTTATTACAGTGGCAATGAGGATG
COL2A1	TGGTGGAGCAGCAAG
COL1A1	CACTGGTGATGCTGGTCCTG
ACAN	ACTGGCGAGCACTGTAAC
SOX9	CAGTACCCGCACTTGCACA
TIMP1	AATTCCGACCTCGTCATCAG
TIMP2	GTAGTGATCAGGGCCAAAGC
TIMP3	GTACCGAGGCTTCACCAAGA
SDHA	TGGGAACAAGAGGGCATCTG
MMP1	CTGGAATTGGCCACAAAGTT
MMP3	GGTGTGGAGTTCCTGATGTTG
MMP13	GTGGTGATGAAGATGATT
LGALS1	ATGGCTTGTGGTCTGGTC
LGALS3	GTGCCTTATAACCTGCCTTTG
LGALS8	AACCCTGACGGCACTTAGC
	**reverse**
IL1B	AGTGGTGGTCGGAGATTCG
COL2A1	GGGAGGCGTGAGGTCTTCTG
COL1A1	CGAGGTCACGGTCACGAAC
ACAN	TCTTGGGCATTGTTGTTGAC
SOX9	CCCGTTCTTCACCGACTTCC
TIMP1	GTTGTGGGACCTGTGGAAGT
TIMP2	GGGGGCCGTGTAGATAAACT
TIMP3	ACCTCTCCACGAAGTTGCAC
SDHA	CCACCACTGCATCAAATTCATG
MMP1	TCCTGCAGTTGAACCAGCTA
MMP3	AGCCTGGAGAATGTGAGTGG
MMP13	TGTAGGATGGTAGTATGAT
LGALS1	AGGTTGTTGCTGTCTTTGC
LGALS3	GACTCTCCTGTTGTTCTCATTG
LGALS8	GGATTGAAACTGAGGCACTGG
Probe sequence	no probes used
In silico	All primers were subjected to BLAST analysis
empirical	Primers were used at 100 nM
Priming conditions	For cDNA synthesis, random RT primers were used as provided by the manufacturer (High Capacity cDNA Reverse Transcription Kit (Applied Biosystems, Austria)).
PCR efficiency	qPCR efficiencies were determined using dilution series of cDNA prepared from chondrocyte mRNA. Used efficiencies are given below in %.
IL1B	95.1
COL2A1	92.4
COL1A1	89.2
ACAN	98.6
SOX9	100.0
TIMP1	100.3
TIMP2	101.9
TIMP3	95.9
SDHA	100.0
MMP1	93.9
MMP3	99.9
MMP13	96.7
LGALS1	101.6
LGALS3	98.3
LGALS8	95.5
Linear dynamic range	The dynamic range of the primers was spanning unknown targets as determined by dilution curves.
Limits of detection	LODs were not determined in this study.
Intra-assay variation	The intra-assay variation was below one Cq.
**RT/PCR**	
Protocols	RT: equal RNA quantities were reverse transcribed into cDNA using the High Capacity cDNA Reverse Transcription Kit (Applied Biosystems, Austria; LOT#: 00810836).<br>qPCR: reactions were performed in 25 µl reaction mixtures containing 1 µl cDNA, 12.5 µl SensiMix SYBR Green Master Mix (Bioline, Austria; LOT#: *S*M605-B060900), 100 nM primers (Metabion, Martinsried, Germany), and nuclease-free water to 25 µl, and run in duplicate on a QuantStudio3 QPCR system (Applied Biosystems).
Reagents	see above.
Duplicate RT	Replicates of the RT step were not performed in this study.
NTC	NTCs were included in each run. The absence of both amplification and melting curve peaks were mandatory for the acceptance of the run.
NAC	Minus RT controls were prepared from randomly selected RNA samples.
Positive control	Inter-run calibrators were not used.
**Data analysis**	
Specialist software	Data were analyzed using the QuantStudio3 software (Applied Biosystems) considering both amplification efficiencies and normalization to SDHA as reference gene.
Statistical justification	For each experiment, chondrocytes were isolated from 3-6 donors and cultured and analyzed separately. All qPCR reactions were performed in duplicate. Data were exported to IBM SPSS v25 and analyzed using the integrated statistic tools. p-values < 0.05 were considered significant.
Transparent, validated normalization	Among a selection of 6 candidate reference genes (please see doi:10.1186/1471-2199-8-13 for details on sequences and primer efficiencies), SDHA was selected as stable reference gene for experiments with primary chondrocytes under the experimental conditions of this study.
