# Supplementary figures and images for: The Dysregulated Galectin Network Activates NF-κB to Induce Disease Markers and Matrix Degeneration in 3D Pellet Cultures of Osteoarthritic Chondrocytes
Source: Calcif Tissue Int. 2020 Nov 13;108(3):377–90. doi: 10.1007/s00223-020-00774-4 (PMC7881967; doi:10.1007/s00223-020-00774-4)

**A**

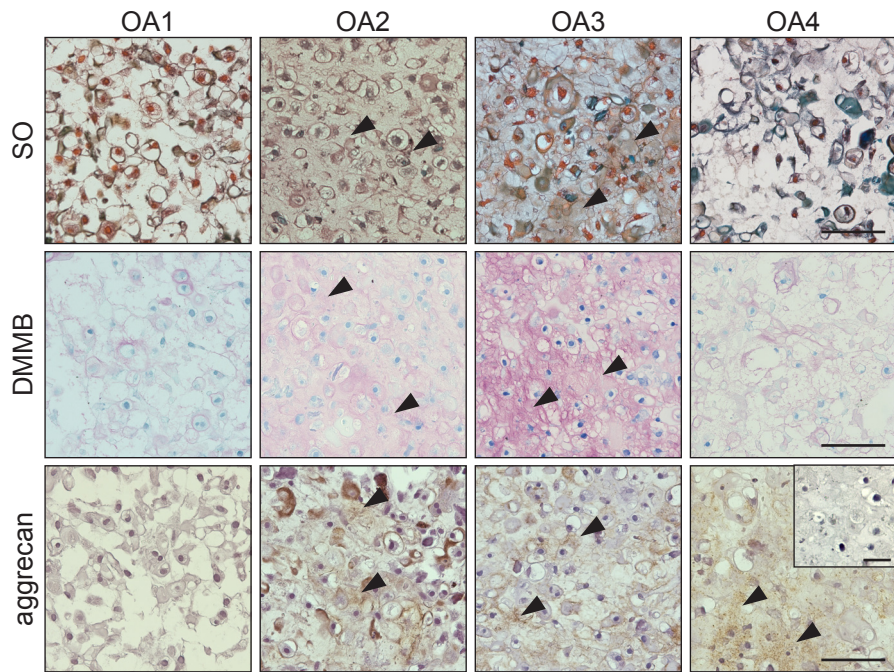

Supplement: Supplementary file 3 — Supplemental file 3 Consecutive histological sections of OA chondrocyte pellets from four patients (OA1-4) were stained with SO, DMMB, or immunohistochemically stained for aggrecan (3 technical replicates). Arrowheads indicate examples of positivity. Representative negative control for immunohistochemical staining is added as inset to OA4. Scale bars: 50 µm (main images, exemplarily depicted in OA4), 20 µm (inset). (PDF 778 kb) [file 223_2020_774_MOESM3_ESM.pdf]

# Supplemental file 4.

**A**

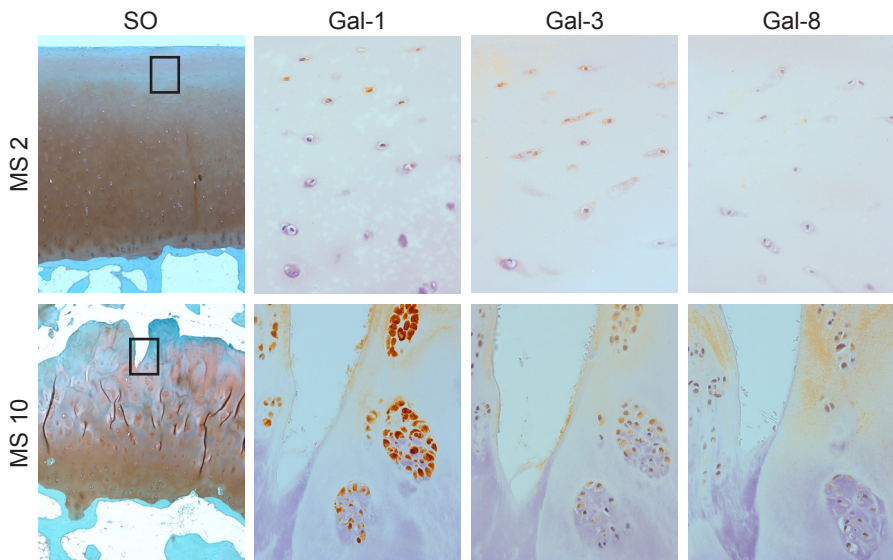

**B**

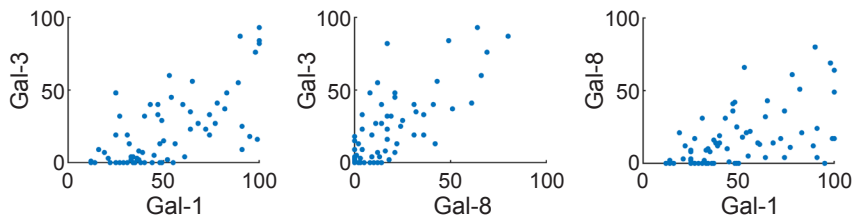

**C**

|                 | Spearman | p-value               |
|-----------------|----------|-----------------------|
| Gal-1 vs. Gal-3 | 0.66     | $6.7 \times 10^{-10}$ |
| Gal-1 vs. Gal-8 | 0.56     | $3.5 \times 10^{-7}$  |
| Gal-3 vs. Gal-8 | 0.72     | $3.4 \times 10^{-12}$ |

Supplement: Supplementary file 4 — Supplemental file 4 Cartilage degeneration is accompanied by the increase of chondrocyte positivity for Gal-1, -3 and -8 measured immunohistochemically. (a) Consecutive sections of articular knee cartilage from 10 OA patients were stained with Safranin O (SO) and immunohistochemically stained for Gal-1, Gal-3, or Gal-8 (3 technical replicates). Shown is a series of stainings of pellets from one representative patient. MS 2 = Mankin score 2; MS 10 = Mankin score 10. Scale bars: 200 µm (SO staining), 20 µm (galectin stainings). (b) Correlation analyses of Gal-1, Gal-3 and Gal-8 shown as pairwise scatter plots for 70 measurements (3 technical replicates). (c) Statistical outcome of correlation analyses between the positivities for the three galectins including Spearman correlation coefficients and p-values. (PDF 426 kb) [file 223_2020_774_MOESM4_ESM.pdf]

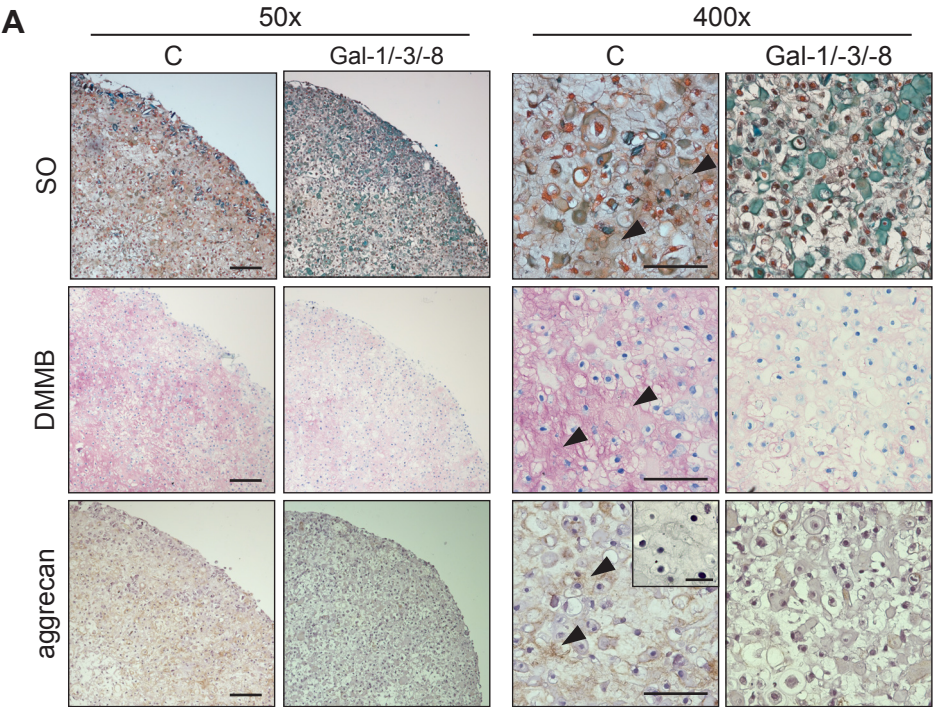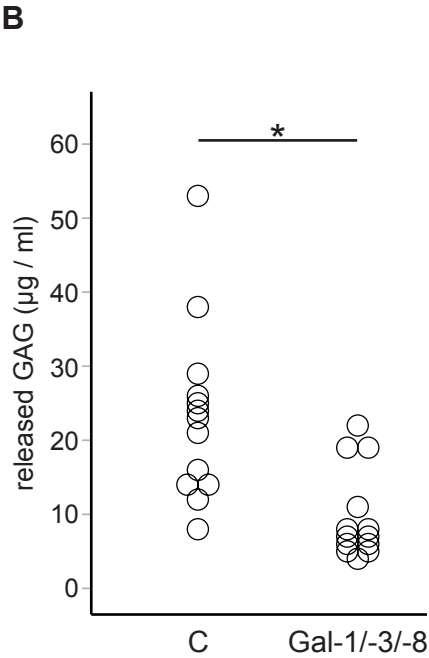

Supplement: Supplementary file 6 — Supplemental file 6 (a) OA chondrocyte pellets from four donors were cultured for three weeks followed by two weeks of treatment with or without Gal-1/-3/-8 (5/1/5 µg/ml). Consecutive histological sections of OA chondrocyte pellets were stained with SO, DMMB, or with anti-aggrecan antibodies (3 technical replicates; left panel, 50x magnification; right panel, 400x magnification). Shown is a series of stainings of pellets from one representative patient (i.e., OA3 in Supplementary file 4). Scale bars (exemplarily depicted in images of untreated control pellets): 200 µm (50x), 50 µm (400x). (b) OA chondrocyte pellets from 13 donors were cultured for three weeks followed by two weeks of treatment with or without Gal-1/-3/-8 (5/1/5 µg/ml). Supernatants of pellets were evaluated for the presence of GAGs (µg/ml) using the DMMB method. Significant differences between groups are indicated with asterisk (*p<0.05, n=13, Wilcoxon test). (PDF 851 kb) [file 223_2020_774_MOESM6_ESM.pdf]
