# Supplementary material for: The Dysregulated Galectin Network Activates NF-κB to Induce Disease Markers and Matrix Degeneration in 3D Pellet Cultures of Osteoarthritic Chondrocytes
Source: Calcif Tissue Int. 2020 Nov 13;108(3):377–90. doi: 10.1007/s00223-020-00774-4 (PMC7881967; doi:10.1007/s00223-020-00774-4)
Supplement: Supplementary file 5 — Supplemental file 5 Comparison of the sets of proteoglycan-related regulated by Gal-1, Gal-3 or Gal-8S as determined by microarray analysis. The ratios between mRNA levels of genes in Gal-1, Gal-3 or Gal-8S-treated versus untreated chondrocytes as well as p-values, corrected for multiple hypothesis testing by the Benjamini–Hochberg method, are given. Data reproduced from GEO (accession numbers: Gal-1: GSE68760, Gal-3: GSE85254, Gal-8S: GSE131822). (PDF 63 kb) [file 223_2020_774_MOESM5_ESM.pdf]

GENE	FUNCTION	ratio treated/ untreated	adj. p-Val	ratio treated/ untreated	adj. p-Val	ratio treated/ untreated	adj. p-Val
Protein degradation							
MMP10	Degrades fibronectin, gelatins of type I, III, IV, and V	10.6	5.E-04	8.0	1.E-03	12.5	3.E-06
MMP12	Hydrolysis of soluble and insoluble elastin.	15.8	7.E-05	8.3	7.E-04	12.4	3.E-07
MMP13	Degradation of fibrillar collagen, fibronectin, TNC and ACAN.	1.3	3.E-02	4.7	2.E-04	8.3	2.E-04
MMP1	Cleaves collagens of types I, II, and III.	1.5	4.E-01	5.0	3.E-03	7.5	6.E-04
MMP7	Degrades casein, gelatins of types I, III, IV, and V, and fibronectin.	1.4	5.E-02	2.6	3.E-02	2.7	7.E-03
MMP16	Cleaves fibronectin and also collagen type III.	0.7	2.E-03	0.7	2.E-02	0.7	7.E-04
MMP9	Cleaves type IV and type V collagen as well as fibronectin.	3.4	2.E-03	1.4	4.E-01	0.6	3.E-04
MMP19	Degrades aggrecan and cartilage oligomeric matrix protein.	2.3	2.E-04	1.3	2.E-01	1.2	3.E-01
ADAMTS9	Cleaves the large aggregating proteoglycans, aggrecan and versican.	2.6	9.E-05	4.5	3.E-03	4.6	8.E-07
ADAMTS4	Cleaves aggrecan.	6.4	4.E-03	3.2	4.E-02	2.4	5.E-02
ADAMTS12	Cleaves alpha-2 macroglobulin and aggregan.	0.8	4.E-02	0.9	5.E-01	0.7	2.E-02
ADAMTS13	Cleaves the von Willebrand factor.	0.6	2.E-04	0.7	7.E-03	0.7	2.E-03
ADAMTS1	Cleaves aggrecan.	0.1	3.E-04	0.6	4.E-01	0.5	2.E-01
Core proteins							
ASPN	Asporin binds collagen, competing for the same binding sites as decorin.	0.1	8.E-06	0.1	2.E-03	0.1	5.E-06
OGN	Widely expressed in connective tissues, including cartilages, with unclear function.	0.2	6.E-06	0.5	2.E-02	0.3	2.E-03
SDC1	Cell surface proteoglycan that bears both heparan sulfate and chondroitin sulfate	0.2	2.E-06	0.3	3.E-03	0.3	2.E-04
MATN2	Involved in cartilage matrix assembly.	0.2	2.E-04	0.5	3.E-01	0.4	9.E-02
DCN	May affect the rate of fibrils formation.	0.2	4.E-04	1.2	6.E-01	0.5	8.E-02
HAPLN1	Stabilizes the aggregates of proteoglycan monomers with hyaluronic acid in the extracellular cartilage matrix.	0.2	7.E-04	0.8	6.E-01	0.5	8.E-02
GPC6	Cell surface proteoglycan that bears heparan sulfate.	0.3	2.E-05	0.6	4.E-02	0.5	7.E-03
MATN3	Major component of the extracellular matrix of cartilage and may play a role in the formation of extracellular filamentous networks.	0.3	1.E-02	0.6	3.E-01	0.4	4.E-02
VCAN	May play a role in intercellular signaling and in connecting cells with the extracellular matrix.	0.4	3.E-02	0.5	2.E-01	0.6	2.E-01
FMOD	Affects the rate of fibrils formation. May have a primary role in collagen fibrillogenesis.	0.5	3.E-04	0.8	3.E-01	0.7	7.E-02
ACAN	Major component of extracellular matrix of cartilagenous tissues	0.6	5.E-02	0.5	3.E-02	0.3	8.E-05
GPC3	Cell surface proteoglycan that bears heparan sulfate.	1.1	3.E-01	1.1	5.E-01	1.2	8.E-02
HAPLN3	May function in hyaluronic acid binding.	1.1	3.E-01	1.7	2.E-05	1.9	1.E-05
SDC4	Cell surface proteoglycan that bears heparan sulfate	3.9	7.E-06	3.2	6.E-04	3.3	5.E-05
Glycosaminoglycan chain polymerisation							
EXTL2	Glycosyltransferase required for the biosynthesis of heparan-sulfate.	0.3	2.E-05	0.7	5.E-02	0.6	2.E-03
CHSY1	Transfers GlcUA and GalNAc to the non-reducing end of the elongating chondroitin polymer.	0.5	5.E-05	0.6	2.E-02	0.8	7.E-02
CHSY3	Transfers GlcUA and GalNAc to the non-reducing end of the elongating chondroitin polymer.	0.5	2.E-03	0.9	6.E-01	0.7	6.E-02
EXTL1	Probable glycosyltransferase.	0.9	3.E-01	0.7	5.E-02	0.6	6.E-03
CSGALNACT1	Required for elongation of chondroitin chains.	1.8	2.E-03	1.5	4.E-02	1.4	5.E-02
HAS3	Catalyzes the addition of GlcNAc or GlcUA monosaccharides to the nascent hyaluronan polymer.	2.8	4.E-04	1.5	2.E-01	1.3	2.E-01
HAS2	Catalyzes the addition of GlcNAc or GlcUA monosaccharides to the nascent hyaluronan polymer.	3.4	5.E-03	1.6	2.E-01	1.8	3.E-02
HAS1	Catalyzes the addition of GlcNAc or GlcUA monosaccharides to the nascent hyaluronan polymer.	3.8	2.E-05	2.2	1.E-01	2.1	9.E-02
Glycosaminoglycan chain modification							
SUMF1	Catalyzes the conversion of cysteine to 3-oxoalanine on target proteins.	0.3	6.E-05	0.5	6.E-02	0.5	1.E-02
CHST14	Catalyzes the transfer of sulfate to position 4 of the GalNAc residue of dermatan sulfate.	0.4	2.E-04	0.7	2.E-01	0.7	1.E-01
CHST6	Catalyze the transfer of sulfate to position 6 of non-reducing GlcNAc residues of keratan.	0.4	1.E-04	0.6	5.E-02	0.6	1.E-02
NDST1	Catalyzes both the N-deacetylation and the N-sulfation of GlcNAc of the glycosaminoglycan in heparan sulfate.	0.4	2.E-04	0.7	2.E-01	0.6	7.E-02
SUMF2	Inhibits the activation of sulfatases by SUMF1.	0.4	2.E-04	0.7	2.E-01	0.6	6.E-02
HS3ST1	Catalyzes the transfer of a sulfo group to position 3 of glucosamine residues in heparan.	0.5	2.E-02	0.4	1.E-02	0.6	7.E-02
CHST1	Catalyze the transfer of sulfate to position 6 of Gal residues of keratan.	0.6	2.E-04	0.7	9.E-03	0.6	2.E-04
HS6ST2	Catalyzes the transfer of sulfate to position 6 of the GlcNS of heparan sulfate.	0.7	6.E-03	0.8	2.E-01	0.9	4.E-01
CHST15	Transfers sulfate to chondroitin sulfate A and forms chondroitin sulfate E containing GlcA-GalNAc(4,6-SO(4)) repeating units.	1.1	8.E-01	1.8	5.E-02	1.8	2.E-02
CHST9	Catalyzes the transfer of sulfate to position 4 of non-reducing GalNAc residues. Isoform 2 is active towards chondroitin.	1.2	2.E-02	1.0	9.E-01	1.1	2.E-01
CHST7	Catalyzes the transfer of sulfate to position 6 of GalNAc residue of chondroitin.	2.2	6.E-05	1.5	6.E-02	1.8	2.E-03
HS3ST3B1	Catalyzes the transfer of a sulfo group to an N-unsubstituted glucosamine linked to a 2-O-sulfo iduronic acid unit on heparan sulfate.	3.4	6.E-05	2.1	2.E-02	3.3	2.E-04
CHST11	Sulfotransferase that transfers sulfate to position 6 of Gal residues of keratan.	5.7	8.E-07	5.1	4.E-05	4.6	8.E-06
CHST2	Sulfates GlcNAc residues at terminal, non-reducing ends of oligosaccharide chains.	7.6	2.E-05	4.4	3.E-03	7.0	5.E-05
Glycosaminoglycan chain degradation							
HYAL1	Hydrolysis of (1->4)-linkages between N-acetyl-beta-D-glucosamine and D-glucuronate residues in hyaluronate.	0.3	1.E-04	0.6	7.E-02	0.7	5.E-02
ARSK	Arylsulfatase activity.	0.4	1.E-05	0.6	4.E-02	1.0	8.E-01
SGSH	Catalyzes a step in lysosomal heparan sulfate degradation.	0.7	2.E-03	0.7	6.E-02	0.6	2.E-03
ARSJ	Arylsulfatase activity.	1.8	1.E-04	1.5	1.E-02	1.4	2.E-02
